# Supplementary material for: LGR5 Expression Predicting Poor Prognosis Is Negatively Correlated with WNT5A in Colon Cancer
Source: Cells. 2023 Nov 20;12(22):2658. doi: 10.3390/cells12222658 (PMC10670301; doi:10.3390/cells12222658)
Supplement: Supplementary file 1 [file cells-12-02658-s001.zip › cells-2669657-supplementary.pdf]

Supplementary Information

LGR5 expression predicting poor prognosis is negatively correlated with WNT5A in colon cancer

Lubna M. Mehdawi [†,*], Souvik Ghatak[†], Payel Chakraborty, Anita Sjölander and Tommy Andersson*

Cell and Experimental Pathology, Department of Translational Medicine, Lund University, Skåne University Hospital, Malmö, Sweden.
* Correspondence: tommy.andersson@med.lu.se (T.A.); lubna.mehdawi@med.lu.se (L.M.)
[†] These authors contributed equally to this study.

cohort.

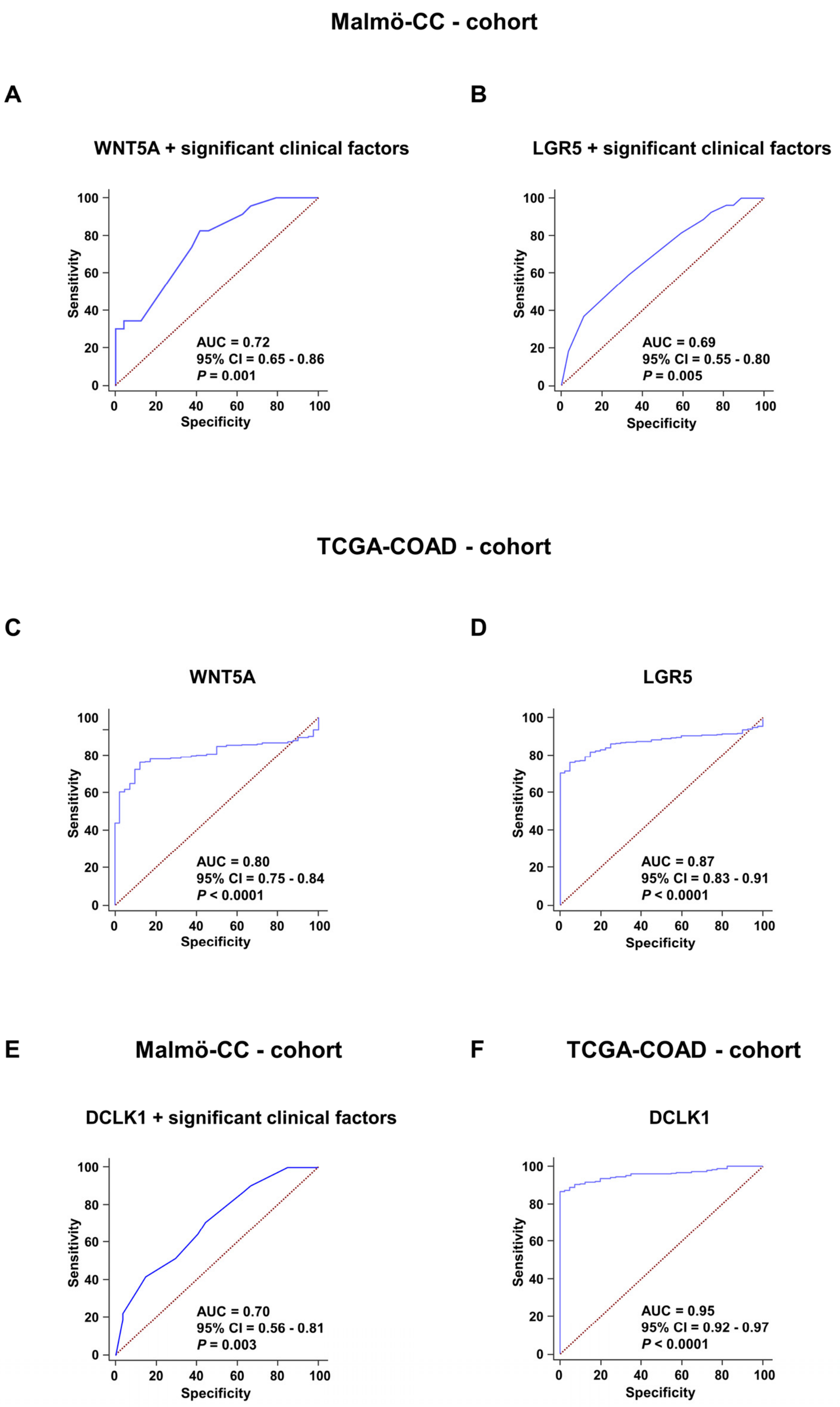

Figure S1. ROC curves to determine the predictive ability of **(A)** WNT5A, **(B)** LGR5, and **(E)** DCLK1 expression in the Malmö-CC cohort and **(C)** *WNT5A*, **(D)** *LGR5*, and **(F)** *DCLK1* in the TCGA-COAD cohort.

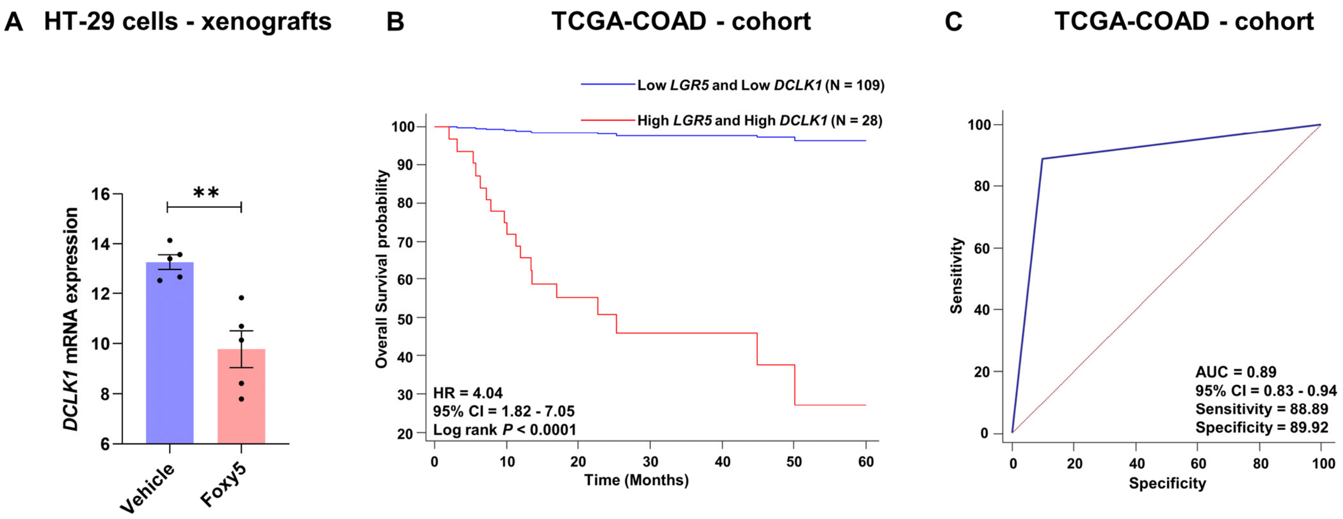

Figure S2. (A) *DCLK1* mRNA levels in HT-29 colon cancer tissues from xenograft mice treated with vehicle or Foxy5 (2 µg/g) every other day for a 14-day period. The results are shown as the mean ± SEM; **P > 0.01, analyzed with paired Student's t test. **(B)** Five-year overall survival analysis of two patient groups from the TCGA-COAD cohort based on the combinations of *LGR5* and *DCLK1* mRNA expressions. **(C)** A ROC curve to determine the predictive ability of the combination of *LGR5* and *DCLK1* expression levels in the TCGA-COAD cohort.

Table S1. The clinicopathological parameters of the patients included in the Malmö-CC cohort at their time of diagnosis. The univariate Cox-PH survival analysis was performed for the listed clinicopathological parameters. However, a univariate Cox-PH analysis was not performed in the T1 to T4 group due to only 1 sample in the T4 group. Instead, we performed a univariate Cox-PH analysis between the low aggressiveness group (T1 + T2) and the high aggressiveness group (T3 and T4).

Clinicopathological parameters	Patients (n = 72)	Percentage (%)	HR (95% CI)	P value
**Sex**				
Male	39	54.16	2.00 (1.10–3.61)	0.024
Female	33	45.83		
**Age (75 ± 9.94)**				
< 75	33	45.83	0.99 (0.96–1.03)	0.960
≥ 75	39	54.16		
**Pathological stage**				
Stage I	9	12.50	2.72 (1.85–4.01)	< 0.0001
Stage II	28	38.88		
Stage III	21	29.16		
Stage IV	14	19.44		
**T Group**				
T1	3	4.16	NA*	NA
T2	9	12.50		
T3	59	81.94		
T4	1	1.38		
**T Group**				
T1 and T2	12	16.66	2.37 (0.85-6.60)	0.100
T3 and T4	60	83.33		
**Lymph node metastasis**				
N0	40	55.55	2.28 (1.61–3.24)	< 0.0001
N1	27	37.50		
N2	2	2.77		
N3	3	4.16		
**Metastasis**				
M0	58	80.55	6.09 (3.00–12.35)	< 0.0001
M1	14	19.44		
**Tumor size**				
0-2 cm	2	2.77	2.84 (0.48–5.37)	0.083
2-5 cm	10	13.88		
5-10 cm	46	63.88		
>10 cm	9	12.50		
Missing	4	5.55		
**Tumor differentiation**				
Poor	2	2.77	0.93 (0.51–1.68)	0.821
Moderate	50	69.44		
High	20	27.77		

NA* = Not Applicable; HR – Hazard Ratio; CI – Confidence Interval.
